# Immunoexpression of Spexin in Selected Segments of the Bovine (*Bos taurus taurus*) Gastrointestinal Tract

**DOI:** 10.3390/ani13243789

**Published:** 2023-12-08

**Authors:** Aleksandra Dajnowska, Cezary Osiak-Wicha, Małgorzata Piech, Siemowit Muszyński, Ewa Tomaszewska, Katarzyna Ropka-Molik, Michał K. Krzysiak, Marcin B. Arciszewski

**Affiliations:** 1 Department of Animal Anatomy and Histology, Faculty of Veterinary Medicine, University of Life Sciences in Lublin, Akademicka 12, 20-950 Lublin, Poland; aleksandra.dajnowska@up.lublin.pl (A.D.); cezary.wicha@up.lublin.pl (C.O.-W.); malgorzata.piech@up.lublin.pl (M.P.); 2 Department of Biophysics, Faculty of Environmental Biology, University of Life Sciences in Lublin, Akademicka 13, 20-950 Lublin, Poland; siemowit.muszynski@up.lublin.pl; 3 Department of Animal Physiology, Faculty of Veterinary Medicine, University of Life Sciences in Lublin, Akademicka 12, 20-950 Lublin, Poland; ewarst@interia.pl; 4 Department of Animal Molecular Biology, National Research Institute of Animal Production, Krakowska 1, 32-083 Balice, Poland; katarzyna.ropka@iz.edu.pl; 5 Białowieża National Park, Park Pałacowy 11, 17-230 Białowieża, Poland; michal.krzysiak@bpn.com.pl; 6 Institute of Forest Sciences, Faculty of Civil Engineering and Environmental Sciences, Białystok University of Technology, Wiejska 45 E, 15-351 Białystok, Poland

**Keywords:** cow, spexin, ruminants, ENS, *GALR2*

## Abstract

**Simple Summary:**

Our research delved into the exploration of spexin, a neuropeptide, within the stomach, small intestine, and colon of cows. While the roles of spexin in smaller animals are established, our focus was to bridge the knowledge gap regarding its functions in larger mammals. Through the analysis of selected segments of the unique gastrointestinal tract of the cattle, we sought to unravel the locations of spexin in the gastrointestinal tract of this polygastric animal. Remarkably, our findings revealed heightened gene expression of a specific spexin receptor, galanin receptor type 2, in the small intestine. This finding provides valuable insights into the functioning of spexin in larger livestock animals, particularly in terms of digestive regulation. The implications extend beyond veterinary considerations, potentially providing a window into understanding digestive processes in humans. This study enhances our overall understanding of neuropeptide functions and establishes a foundation for progress in animal health research.

**Abstract:**

In the expansive domain of neuropeptide investigation, spexin (SPX) has emerged as a captivating subject, exerting a significant impact on diverse physiological processes. Initially identified in mice, SPX’s distribution transcends various organs, suggesting its potential regulatory roles. Despite extensive research in smaller species, a notable gap exists in our comprehension of SPX in larger mammals, particularly ruminants. Our study meticulously explores the immunolocalization of SPX within the gastrointestinal organs of bovines, with a specific focus on the abomasum, jejunum, and colon. Tissue samples from Holstein–Friesian cattle underwent careful processing, and gene mRNA expression levels, particularly *GALR2* and *SPX*, were assessed. Intriguingly, our findings revealed that *GALR2* expression was highest in the jejunum, signifying a potentially critical role in this digestive segment. Immunohistochemistry further unveiled distinct patterns of SPX immunoreactivity in each examined region—abomasum, jejunum, and colon—highlighting nuanced, region-specific responses. Notably, the abomasum and jejunum predominantly exhibited positive immunoreactivity in the submucosal plexus, while the colon, in contrast, demonstrated a higher degree of immunoreactivity in myenteric plexus neurons. Our investigation, grounded in the hypothesis of ubiquitous SPX distribution in ruminants, delves deeper into the intricate role of SPX within the enteric nervous system. This study meticulously explores the spatial distribution of SPX within the myenteric and submucosal plexuses, integral components of the enteric nervous system. These findings significantly enhance our understanding of SPX’s potential roles in gastrointestinal regulation in bovines, providing a unique perspective on larger mammals and enriching our comprehension of this intriguing neuropeptide’s significance in various physiological processes.

## 1. Introduction

Within the realm of neuropeptide inquiry, spexin (SPX) emerges as an intriguing subject of study. Comprising a modest 14 amino acids, the discovery of SPX in 2007 was facilitated through the application of bioinformatics tools, specifically a hidden Markov model [1,2,3,4]. This neuropeptide, a member of the SPX/galanin/kisspeptin gene family [5,6], has been identified as a natural ligand for galanin receptors 2 and 3 (GALR 2/**3**) [7]. Its far-reaching impact on a multitude of physiological processes throughout the organism, combined with its extensive distribution, has evoked significant scientific interest [8].

The initial unveiling of SPX took place within the submucosal layer of the esophagus and the stomach fundus in mice [4]. Building upon this foundational revelation, subsequent studies led by Porzionato et al. [9] expanded our understanding of SPX’s distribution and effects. Utilizing reverse transcription-polymerase chain reaction (RT-PCR) and immunohistochemistry (IHC), these research efforts revealed the presence of SPX in various tissues in rats [10,11]. SPX was found to be widespread across various organs, including the esophagus, stomach, small intestine, liver, pancreas, lung, skeletal muscle, heart, uterus, thymus, spleen, kidney, urinary bladder, brain, hypothalamus, adenohypophysis, thyroid, adrenal gland, testis, and ovary [12,13,14,15,16]. This wide distribution implies the intricate role played by SPX in regulating an assortment of physiological functions, spanning the realms of the endocrine system, food intake, and energy metabolism, including the intricate orchestration of carbohydrates and lipids [17]. Beyond these vital processes, SPX’s influence extends into the domains of reproductive physiology, gastrointestinal processes, cardiovascular dynamics, and nociception. However, despite accumulating evidence of its manifold effects, the precise mechanisms through which SPX exerts its influence remain veiled in theoretical suppositions.

This exceptional neuropeptide, with its broad influence, calls for thorough investigation across various tissues and animal species, underscoring its evolutionary importance and potential role in regulating diverse physiological and pathological processes. Notably, a SPX paralogous gene, known as *SPX-2*, has been recognized in the chromosomal structures of vertebrates [18]. It is important to highlight that this counterpart is notably absent within the genomes of mammals. While substantial efforts have been channeled into deciphering SPX’s enigma in fish, rodents, mice, and humans [18,19,20,21], a discernible lacuna still exists in our comprehension of its presence and ramifications in larger mammals.

Our investigation is grounded in the notion that SPX exhibits a ubiquitous distribution within ruminants’ gastrointestinal organs, suggesting a pivotal role in orchestrating diverse physiological processes within these vital systems. We selected the abomasum (stomach), jejunum (small intestine), and colon (large intestine) as representative samples of individual sections of the bovine gastrointestinal tract (GIT). All mammal species possess an inherent system to control their food consumption, yet this mechanism is not identical among them. Diverse elements, including specific nutritional requirements, breeding systems, and evolutionary developments, contribute to these variations [22,23].

We anticipate that our study will unveil the immunolocalization of SPX within the cellular landscape of these bovine organs, providing empirical substantiation for its presence in these regions. Additionally, based on existing literature, we hypothesize that GALR2 receptors would exhibit immunoexpression within the neuronal populations of the myenteric plexus (MP) and submucosal plexus (SP), which are integral components of the enteric nervous system (ENS), as well as surrounding structures such as the epithelium, glands, and muscles. By exploring the spatial distribution of these receptors within the ENS, our study aims to contribute to a deeper understanding of their potential functional roles in gastrointestinal regulation and their implications for bovine health.

## 2. Materials and Methods

### 2.1. Animals and Tissue Processing

Tissue samples were procured from male Holstein–Friesian breed cattle, aged between 20 and 24 months (*n* = 6), from a local cattle slaughterhouse. Sections of the digestive tract, specifically the abomasum, jejunum, and colon, were promptly excised within 15 min following the animals’ deaths. Subsequently, the organs were meticulously rinsed with a physiological saline solution to eliminate extraneous contaminants. For mRNA analyses, sections were snap frozen in liquid nitrogen, then stored at −80 °C. To facilitate IHC investigation, the specimens were fixed in a 4% formaldehyde solution.

Upon completion of the fixation process, the samples underwent thorough rinsing under running water for a duration of 8 h. This extensive rinsing period was essential for the removal of residual formaldehyde from the specimens. Subsequent to this rinsing phase, a gradual dehydration process was employed, wherein the alcohol concentration was incrementally increased, commencing with 70% ethanol. Each ethanol bath was maintained for a duration of one day, with the final step involving two iterations of immersion in 100% ethanol. The embedding procedure entailed incubating the samples in paraffin at 60 °C overnight. The final step involved submerging the samples in melted paraffin, resulting in the creation of paraffin blocks, which were subsequently sectioned at a thickness of 5–6 µm utilizing a rotary microtome (HM 360, Microm, Walldorf, Germany).

### 2.2. Gene Expression Quantification

Intestinal samples underwent RNA extraction employing the PureLink RNA Mini Kit (Invitrogen, Waltham, MA, USA) following the prescribed protocols. To ensure genomic DNA contamination elimination, the isolated RNA underwent treatment with DNase I (PureLink DNase Set; Invitrogen). Subsequently, the NanoDrop 2000 spectrophotometer (Thermo Fisher Scientific, Wilmington, DE, USA) gauged the total RNA concentration and assessed potential protein/chemical contamination, while 2% agarose gel electrophoresis verified RNA integrity. For cDNA synthesis, 250 ng of total RNA underwent transcription using the TranScriba Kit (A&A Biotechnology, Gdańsk, Poland). The resulting cDNA then underwent qPCR to scrutinize *SPX* and *GALR2* gene expression levels. To prevent DNA amplification, primers were designed to target different exons using the Ensembl (http://www.ensembl.org, accessed on 22 November 2023) genome database (Table 1). The qPCR analysis utilized the RT-PCR Mix SYBR Green (A&A Biotechnology, Gdańsk, Poland) on the QuantStudio 7 Flex system (Applied Biosystems, Waltham, MA, USA). Endogenous controls *RPS9* and *ACTB* [24], chosen for their stable expression across all bovine tissue types and experimental conditions, facilitated data normalization. Primer designs for both target and control genes were crafted using Primer3web (https://primer3.ut.ee, accessed on 22 November 2023), with the synthesized primers sourced from Genomed (Genomed, Warszawa, Poland). Triplicate technical replicates analyzed each sample, and the −ΔΔCT cycle threshold method [25] computed the relative expression of *SPX* and *GALR2* genes in the abomasum, jejunum, and colon. All results were normalized based on the expression level of a specific gene in the abomasum. 

### 2.3. Immunohistochemistry

Immunohistochemical staining was executed using a primary rabbit polyclonal antibody, specifically for recognizing SPX in humans, rats, and mice (Table 2). The antibody was diluted in Diamond antibody diluent (Cell Marque Corp., Rocklin, CA, USA). The process commenced with the deparaffinization of sections in xylene for three iterations of 5 min each, followed by hydration in a descending ethanol series (ranging from 100% to 50%) for 2 min each. Subsequently, the sections were rinsed in deionized water for 5 min. To inhibit endogenous peroxidase activity, the sections were immersed in a 3% hydrogen peroxide solution in deionized water at room temperature (RT) for 10 min. Antigen retrieval was conducted in a Rapid Cook pressure cooker (Morphy Richards, Swinton, UK) utilizing a sodium citrate buffer (10 mM sodium citrate, 0.05% Tween 20, pH 6.0), in accordance with the protocols specified by the antibody manufacturer. Following antigen retrieval, the sections were washed twice in PBS for 5 min and then incubated in a blocking serum (UltraVision Protein Block, Thermo Scientific TA-125-PBQ, Kalamazoo, MI, USA) at RT. After 8 min, they underwent a PBS washing step (4 iterations of 5 min each) and were subsequently incubated with the anti-SPX p-Rb primary antibody (Table 2) overnight at 4 °C within a humid chamber. The following day, PBS washing was repeated (4 iterations of 5 min each), and the sections were processed with a ready-to-use, two-step detection system. The first step was incubated for 15 min, followed by a PBS washing step (2 iterations of 5 min each), and the subsequent deposition of the second step for 30 min. The sections were then washed twice with PBS for 5 min. The development of sections was initiated by immersing them in 3,3′Diaminobenzidine (DAB substrate kit, ab64238 Abcam, Cambridge, UK) until a brown coloration was achieved. The reaction was halted by immersing the preparations in tap water three times, followed by three immersions in distilled water. Finally, the sections were counterstained with Mayer’s hematoxylin (Patho, Mar-Four, Konstantynów Łódzki, Poland) for 3 min. Subsequently, the preparations were immersed for 10 min in tap water. After this period, they were briefly immersed in ammonia and then in distilled water. Ultimately, the sections were dehydrated through an ascending series of ethyl alcohol concentrations ranging from 50% to 100%, with each concentration maintained for 10 min. They were subsequently transferred to xylene for 10 min and covered with coverslips. The slides were examined using a light microscope (BX-51 DSU, Olympus, Tokyo, Japan) equipped with a digital color camera (DP-70, Olympus) at magnifications of 10×, 20×, and 40×.

### 2.4. Evaluation of the IHC Reaction Intensity

To assess the intensity of the IHC reaction, color deconvolution was applied in ImageJ software (ver.54f, National Institutes of Health, Bethesda, MD, USA; http://rsb.info.nih.gov/ij/index.html, accessed on 22 November 2023) using the IHC profiler plugin [26]. After deconvolution, DAB images were measured by quantitatively comparing average pixel intensity values on photomicrographs converted to 8-bit grayscale images. The intensity of the immune reaction in each of the analyzed digital images was measured in 10 randomly selected areas of the positive signal in the ENS, epithelium, glands, and muscles. The reaction areas were manually delineated using the freehand selection tool. The staining intensity was determined based on the ‘average grayscale value’ parameter [27]. The grayscale pixel color value ranged from 0 to 255, where 0 was the darkest shade (black pixel) and 255 was the lightest shade (white pixel). Subsequently, the measurements were converted to optical density (OD) using the formula OD = −log(x/255). In this formula, “x” was the measured “mean grey value”. The intensity of the IHC reaction was divided into four categories [28] based on the OD value as follows: strong (>0.6), moderate (0.4–0.6), weak (0.2–0.4), and negative (<0.02).

### 2.5. Statistical Analysis

In order to present the findings, this study utilized mean values accompanied by their respective standard deviations (SD). Prior to statistical analysis, the normal distribution of each variable was assessed employing the Shapiro–Wilk normality test; the homogeneity of variances was assessed using Levene’s test.

Subsequently, data were subjected to a one-way ANOVA followed by Tukey’s HSD post hoc test (mRNA gene expression data) or a two-tailed Student’s *t*-test (to discern differences in the percentage of SPX-positive neurons in SP and MP). For all tests, a *p*-value < 0.05 was established as statistically significant. The entire spectrum of statistical calculations was conducted utilizing GraphPad Prism version 9.5.1 for the Windows operating system (GraphPad Software, San Diego, CA, USA; https://www.graphpad.com, accessed on 22 November 2023).

## 3. Results

### 3.1. Relative Gene Expression Levels

**Figure 1:**
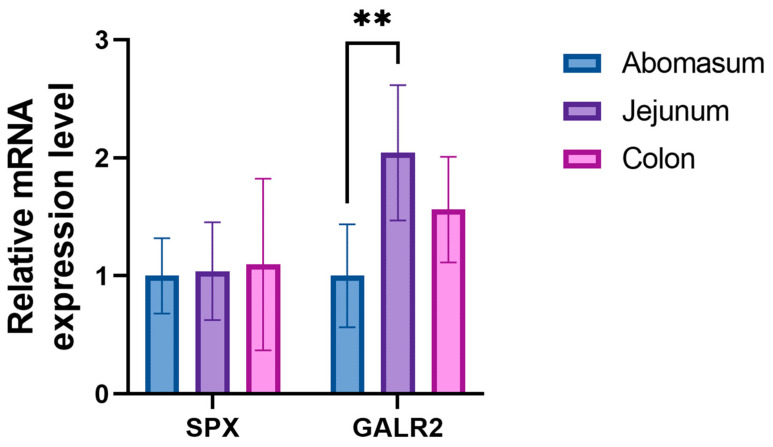
The relative expression of *SPX* and *GALR2* genes in the examined small segments of the bovine gastrointestinal tract (abomasum, jejunum, and colon). Gene expression was normalized by the geometric mean of the *RPS9* and *ACTB* housekeeping genes and presented as relative to the expression level in abomasum. Asterisks (**) indicate significant differences between segments (** *p* < 0.01).

For *SPX*, no significant differences were observed between the segments, as all three exhibit relatively similar mRNA expression levels. The analysis of *GALR2* expression, however, unveiled particularly intriguing results. The jejunum exhibited the highest levels of *GALR2* expression, contrasting notably with the lowest expression observed in the abomasum. Additionally, a significant disparity in mMRA expression was discernible between these distinct sections of the gastrointestinal tract (Figure 1; *p* < 0.01).

### 3.2. Abomasum SPX Immunoreactivity

**Figure 2:**
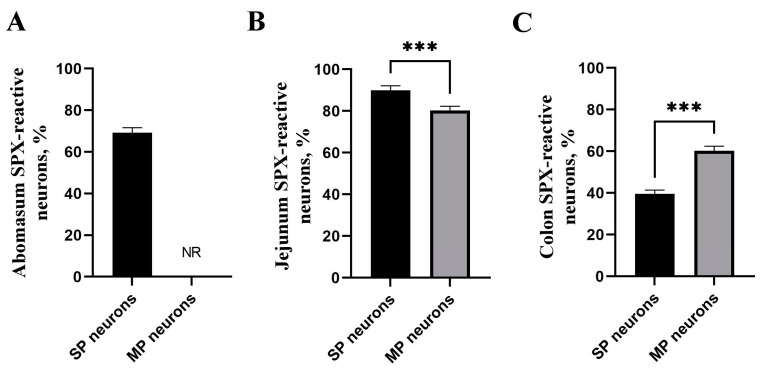
Summary of the percentage of spexin immunoreactive neurons in submucous (SP) and myenteric (MP) plexuses in different sections: (**A**) abomasum; (**B**) jejunum; (**C**) colon of the bovine gastrointestinal tract. Asterisks (***) indicate significant differences in neuron numbers between SP and MP (*** *p* < 0.001).

**Figure 3:**
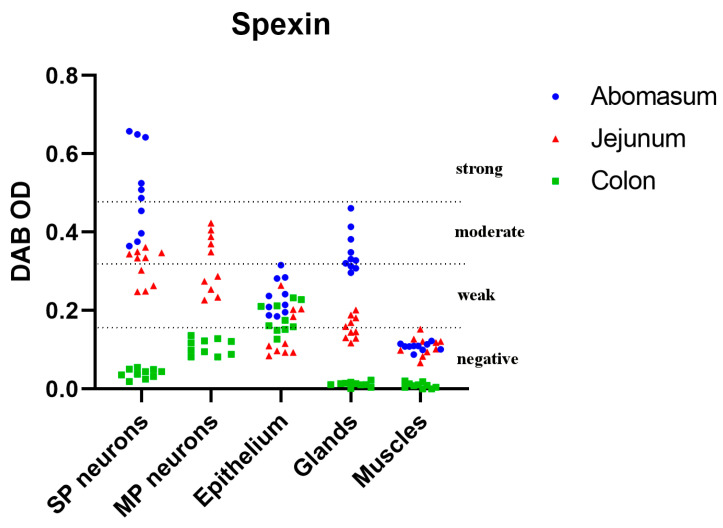
Summary of the intensity of spexin DAB-stained immunoreactivity measured as the optical density (OD) in different cellular structures: submucous plexus (SP) neurons, myenteric plexus (MP) neurons, epithelium, glands, and muscles in the selected sections of the bovine gastrointestinal tract.

**Figure 4:**
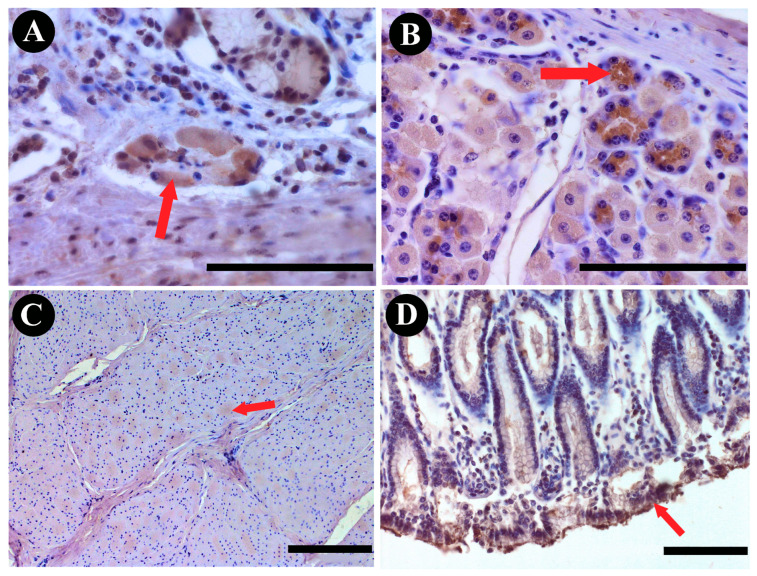
DAB staining of SPX immunoreactivity in bovine abomasum structures: (**A**) submucous plexus; (**B**) glands; (**C**) muscle layers; (**D**) epithelium. Red arrows indicate positive immunoreactivity. Scale bars: 50 µm.

The examination of the immunoreactivity of SPX within the abomasum revealed distinctive patterns. In the SP, the majority of neurons (Figure 2A) displayed a range of immunoreactivity from weak to strong, indicating a substantial presence of SPX. In contrast, MP neurons showed no discernible immunoreactivity, suggesting the absence of SPX within this neuronal subset (Figure 3). Furthermore, the structural components of the abomasum, including the epithelium and glands, demonstrated varying degrees of immunoreactivity. Simple columnar cells in the most superficial epithelium were characterized by a weak reaction, while the base of the gastric glands showed moderate cytoplasmic reactivity. These cells were located between other glandular cells in the basal part of the gastric glands and had contact with the lumen of the gland (Figure 4). The cytoplasm of these cells showed strong staining in the apical part of the cells. Furthermore, SPX immunoreactivity was also observed in parietal cells (in the body and at the base of gastric glands) and foveolar cells (in the neck of gastric pits). In both cases, the cytoplasm showed less staining than the cytoplasm of the chief cells. Additionally, it is noteworthy that the immunoreactivity observed in the abomasum smooth muscle layers, both internal and external, although present, did not meet the criteria for a positive reaction, categorizing the reaction in these structures as negative (Figure 3).

### 3.3. Jejunum SPX Immunoreactivity

**Figure 5:**
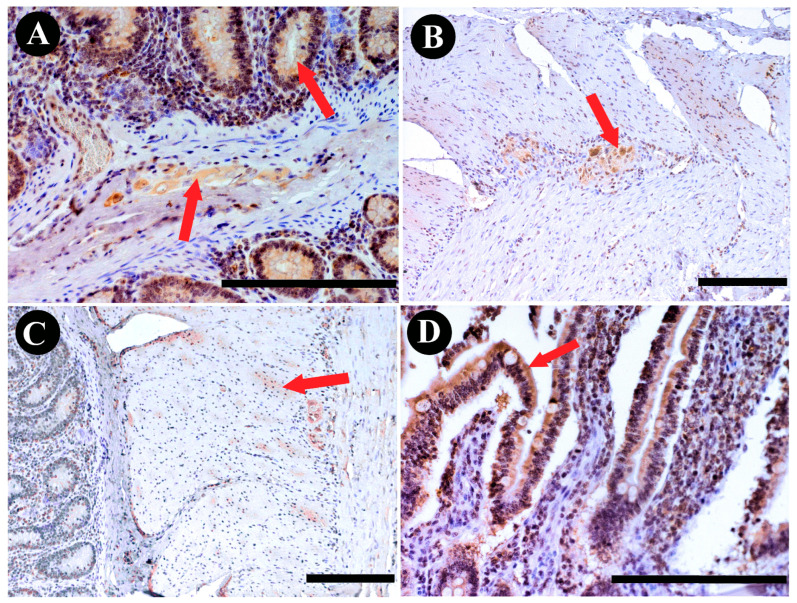
DAB staining of SPX immunoreactivity in bovine jejunum structures: (**A**) submucous plexus and glands; (**B**) myenteric plexus; (**C**) muscle layers; (**D**) epithelium. Red arrows indicate positive immunoreactivity. Scale bars: 50 µm.

In the jejunum, the majority of neurons exhibited positive immunoreactivity (Figure 2), with a notable prevalence of weak reactions (Figure 3). Particularly noteworthy was the observation of significantly greater immunoreactivity in SP neurons when compared to other neuronal subsets (Figure 2; *p* < 0.001). Notably, immunoreactivity was also evident within the structural components of the jejunum, including the epithelium, glands, and muscles (Figure 5). However, based on their OD calculations, the majority of these reactions were rated as negative (Figure 3). Only a small proportion of the epithelium displayed weak immunoreactivity, reflecting a comparatively limited response in these anatomical elements.

### 3.4. Colon SPX Immunoreactivity

**Figure 6:**
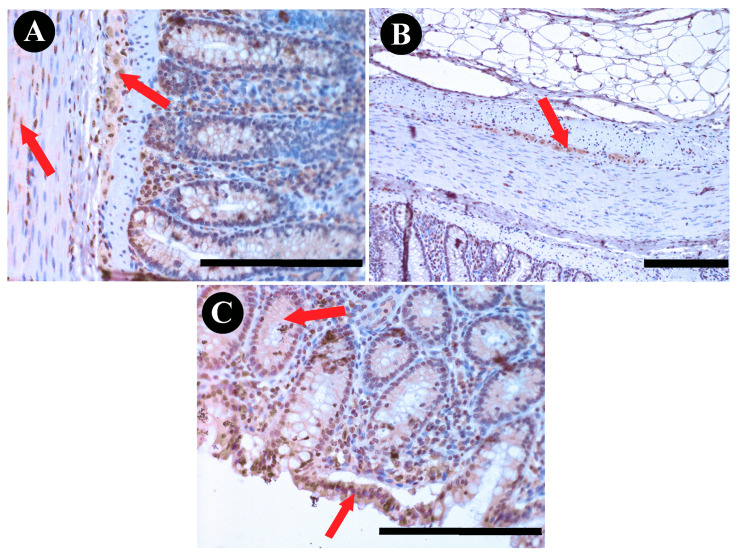
DAB staining of SPX immunoreactivity in bovine colon structures: (**A**) submucous plexus and muscle layers; (**B**) myenteric plexus; (**C**) glands and epithelium. Red arrows indicate positive immunoreactivity. Scale bars: 50 µm.

The examination of immunoreactivity in the colon revealed that the majority of MP neurons exhibited positive immunoreactivity. This response was significantly higher when compared to SP neurons (Figure 2C; *p* < 0.001). Notably, SP neurons showed immunoreactivity in less than half of the neuronal population, highlighting a substantial disparity in the response patterns of these neuronal subsets. Additionally, positive immunoreactivity was also observed within the structural components of the colon, including the epithelium, glands, and muscles (Figure 6). However, the majority of these reactions were notably weak and were categorized as negative (Figure 3). A limited number of reactions in these regions exhibited weak immunoreactivity, indicating a sparse response within the epithelium and glands.

## 4. Discussion

Our exploration of SPX immunoreactivity within the selected segments of bovine GIT has yielded a multitude of findings, shedding light on the intriguing distribution of this neuropeptide and its implications for physiological processes. Our study focused on the abomasum, jejunum, and colon, each of which presented distinct patterns of SPX immunoreactivity, raising pertinent questions about the functional significance of SPX in these regions.

The immunolocalization of SPX within the GIT of dairy cattle remains a less explored domain in current scientific inquiry. Existing studies on SPX have predominantly centered around investigations involving mice, rats, fish, and humans. Thus, the exploration of this neuropeptide in domestic cattle is notably limited. Research by Mikuła et al. [29] previously delved into the role of SPX and other associated metabolites during the transition period in dairy cows. Primarily, this inquiry focused on identifying SPX’s potential as a novel blood marker, offering insights into the health status of animals, particularly during transitional phases. Notably, this study also unveiled the mRNA expression of SPX in various vital organs such as the rumen, intestines, liver, spleen, adipose tissue, skeletal muscles, heart, lungs, kidneys, and spinal cord. This investigation detailed fluctuations in SPX concentration in the blood serum, noting a decrease from 21 days before parturition until the day of parturition, followed by an increase during the initial 14 days of lactation. Intriguing correlations emerged, illustrating negative associations between serum SPX and key metabolites like non-esterified fatty acids, β-hydroxybutyrate, and total ghrelin content. Simultaneously, a positive correlation surfaced between SPX and progesterone levels. These interrelations suggest a potential regulatory role of SPX in the metabolic dynamics of dairy cows. The future trajectory of SPX-related research holds promise for unraveling the complexities surrounding transitional period disorders, particularly those intertwined with lipid metabolism, an area where SPX’s influence is known to be significant.

In the abomasum, a glandular stomach in ruminants, our observations unveiled a conspicuous presence of SPX immunoreactivity within the SP. The majority of SP neurons displayed a range of immunoreactivity from weak to strong, indicating a substantial presence of SPX. Additionally, immunoactivity was evident in the glands; it was negligible in both muscle layers of the stomach and was considered negative. This finding aligns with previous studies in rodents, where increased SPX signals were notably prevalent in the glandular stomach, particularly evident on the surface of the gastric mucosa involving foveolar cells, as well as in the lower mucosal layer characterized by parietal and chief cells constituting the gastric glands; discernible alterations in SPX immunostaining were absent in the muscle layers of both the forestomach and glandular stomach [30]. However, an intriguing divergence was observed within the cows’ MP neurons, where no discernible immunoreactivity was detected. Notably, our findings in the abomasum differ from a study by Ekblad et al. [31] in rats, where immunoreactive fibers were quite numerous in the small intestine, less numerous in the large intestine, and rare in the stomach mucosa. This work was based on galanin, but it has long been shown that SPX acts on galanin receptors [32], so it can be suggested that SPX may exhibit similar behavior. This discrepancy suggests a selective sensitivity to SPX in the submucosal plexus, potentially implying a nuanced role of SPX in regulating specific neuronal subsets within the abomasum.

In the jejunum, SPX immunoreactivity displayed a distinct pattern, with the majority of neurons exhibiting positive immunoreactivity, primarily in the form of weak reactions. Notably, SP neurons displayed a significantly higher degree of immunoreactivity compared to other neuronal subsets. Additionally, our study revealed that the highest mRNA expression of *GALR2* was in the jejunum, while *SPX* gene expression was relatively even across all segments. The higher mRNA expression of *GALR2* in the jejunum and the greater percentage of stained neurons in this segment suggest a potentially higher affinity of SPX for galanin receptors in this region. This finding opens the door to further research into the specific mechanisms through which SPX interacts with its receptors and regulates physiological processes within the gastrointestinal tract. Contrary to our findings, the research conducted by Anselmi et al. [33] on rats revealed an inverse relationship in *GALR2* expression, observing the highest levels in the stomach and the lowest in the small intestine. 

In the colon, a substantial contrast in SPX immunoreactivity patterns was observed. The majority of MP neurons exhibited positive immunoreactivity, with a notably higher degree of response compared to SP neurons. This stark contrast between the response patterns of SP and MP neurons within the colon emphasizes the region-specific sensitivity to SPX within this organ. As in the previous sections, positive immunoreactivity extended to the structural components of the colon, including the epithelium, glands, and muscles. However, a notable portion of these reactions were categorized as negative, with only a limited number displaying weak immunoreactivity. Contrary to the observations made via immunohistochemical staining, the gene expression profiles of both *SPX* and *GALR2* present a notably elevated presence in the colon, a consistency also noted in mouse studies [30].

Consistently, in starved mice, the mRNA of *SPX* was significantly decreased in the intestine and colon [16]. This observation raises an important consideration, as the cows in our study were subjected to a period of fasting before culling, potentially explaining the weak immunoreactivity observed in the colon. Starvation may lead to a reduction in *SPX* expression, affecting the overall response of the colonic tissue to SPX. On the other hand, this study on SPX peripheral effects unveiled its significant role in inhibiting food intake in mice [34]. Similarly, a discovery by Ma et al. [35] points to the presence of a glucose-induced insulin element within fish livers, potentially acting as the postmeal signal connecting food consumption with *SPX* expression, both centrally and within the hepatic domain, which seems to be further confirmed in rats [36]. This anorectic effect, observed during fasting and free-feeding conditions, was mediated through the GALR3 pathway and involved the suppression of neuropeptide Y (NPY) levels in the hypothalamus. In a separate study involving fish [37], a notable upregulation of SPX mRNA expression was observed within the forebrain, distinguishing the postprandial group from the preprandial group. Notably, following a meal, the fed group exhibited a pronounced increase in *SPX* mRNA expression compared to their unfed counterparts. Similarly, an increase in SPX in blood was observed in chickens during a short-term fasting period, with food deprivation triggering the regulatory expression of *SPX*, *GALR2*, and *GALR3* in tissues associated with the metabolism of carbohydrates and lipids [38]. Recent studies also suggest that SPX plays a role in regulating glucose homeostasis. Dai et al. indicate that SPX can significantly influence insulin secretion from pancreatic cells, enhance tissue sensitivity to insulin, and even contribute to increased glucose uptake in the liver and muscles in an insulin-resistant model. The level of SPX decreases during diabetes, and its concentration is correlated with fasting blood glucose levels [39]. Interestingly, as our findings, similar to those of mice, reveal a noteworthy decrease in SPX immunoexpression within the GIT during fasting, this may suggest a dynamic response of these neuropeptides and their receptors to nutritional status, emphasizing the intricate interplay between feeding patterns and the molecular mechanisms involved in metabolic regulation.

The implications of our research may extend beyond the boundaries of the bovine species. Since neuropeptides are pivotal in regulating gut activity, they impact not only the production efficiency of livestock but also influence their health, well-being, and longevity. With such far-reaching implications, studies on neuropeptides, their receptors, and related issues are of great significance [40,41,42]. By bridging the existing gap in our understanding of SPX’s distribution and functional significance within larger mammals, we may unearth insights with broader relevance in the domain of neuropeptide research. As we delve deeper into the intricacies of this enigmatic neuropeptide, our work may hold the potential to not only augment the existing body of knowledge surrounding SPX but also to enhance our understanding of the intricate web of physiological interactions underpinning the health and well-being of animals, with potential implications for human physiology as well [43,44].

## 5. Conclusions

In conclusion, our investigation into SPX immunoreactivity within the gastrointestinal organs of bovines has provided a nuanced understanding of the distribution patterns and potential functional roles of this neuropeptide. The distinct SPX immunoreactivity observed in the abomasum, jejunum, and colon highlights region-specific responses, prompting essential questions about the intricate mechanisms underlying SPX regulation in different parts of the digestive system. Notably, our study may contribute valuable insights to a less-explored area of scientific inquiry, as research on SPX in domestic cattle remains limited compared to smaller animal models. The observed variations in SPX immunoreactivity align with and diverge from findings in rodents, emphasizing the species-specific nature of SPX responses. The elevated mRNA expression of *GALR2* in the jejunum suggests a potential affinity between SPX and galanin receptors in this region, opening avenues for future research into their specific interactions. Furthermore, our observations in the colon, especially in the context of fasting conditions, suggest a dynamic response of SPX and its receptors to nutritional status, implicating their involvement in the intricate interplay between feeding patterns and metabolic regulation.

## Figures and Tables

**Table 1:** Primers used in this study.

Gene	Primer Sequences (5′ to 3′)	Product Length	Ensembl Gene ID
*SPX*	F: TCCTGGTGTTTTCTTTCATGG R: GTCGGAGAGGTCCTTCCTC	152	ENSBTAG00000053951
*GALR2*	F: CGCTGCTTTGCAAGGCGGTGCAC R: AGCTGACGACGAAGGTGAGAGG	304	ENSBTAG00000024910
*ACTB*	F: TCCCTGGAGAAGAGCTACGA R: AGGTAGTTTCGTGAATGCCG	133	ENSBTAG00000026199
*RPS9*	F: CCTCGACCAAGAGCTGAAG R: CCTCCAGACCTCACGTTTGTTC	64	ENSBTAG00000006487

**Table 2:** Primary and secondary antibodies used in this study.

Antibody	Host	Code	Dilution	Source
Primary antibody				
SPX	rabbit	H-023-81	1:200	Phoenix Pharm.
Secondary antibody				
Anti-mouse/rabbit	goat	DPVB-HRP	RTU ^1^	ImmunoLogic

^1^ RTU = ready to use.

## Data Availability

Data are contained within the article.
